# Surgical Interventions for Management of Marcus Gunn Jaw-Winking Phenomenon: A Systematic Review

**DOI:** 10.7759/cureus.82438

**Published:** 2025-04-17

**Authors:** Adham M Musa, Ahmad AlKayyat, Shadan Alrawi, Omer W Atallah, Ali Atoui, Ibrahim Qamhieh, Eman M Ghawanmeh

**Affiliations:** 1 College of Literature, Science, and the Arts, University of Michigan, Ann Arbor, USA; 2 School of Medicine, University of Jordan, Amman, JOR; 3 Medicine, Hashemite University, Zarqa, JOR; 4 Medicine, Jordan University of Science and Technology, Irbid, JOR

**Keywords:** jaw-winking ptosis, marcus gunn jaw-winking syndrome, marcus gunn phenomenon, mgjws, trigemino-oculomotor synkinesis

## Abstract

Marcus Gunn jaw-winking syndrome (MGJWS) is a congenital synkinetic ptosis characterized by involuntary eyelid movement with jaw motion. Various surgical interventions, including levator excision and frontalis suspension, have been employed to improve functional and aesthetic outcomes. However, the optimal approach remains debated due to variable success rates and recurrence risks. This study aims to systematically review the effectiveness of different interventions for MGJWS. A systematic review was conducted following the Preferred Reporting Items for Systematic Reviews and Meta-Analyses (PRISMA), analyzing studies on surgical interventions for MGJWS. Predefined inclusion and exclusion criteria were used. Surgical intervention was found to be effective for the management of MGJWS, but long-term follow-up and additional clinical data are needed to determine optimal techniques and minimize the recurrence risks of the phenomenon.

## Introduction and background

Marcus Gunn jaw-winking syndrome (MGJWS) represents an infrequent congenital form of ptosis that produces eyelid elevation through aberrant trigemino-oculomotor synkinesis when patients perform jaw movements such as chewing or opening their mouth. Marcus Gunn first documented this phenomenon in 1883 when misdirected trigeminal nerve branches to the levator palpebrae superioris muscle resulted in eyelid “jaw-winking” during pterygoid muscle activity [1]. The phenomenon can take place as a result of various different thrusts and movements of the jaw and is not specific to a specific direction or motion [2]. While there have been reports of MGJWS occurring bilaterally, it majorly occurs unilaterally and overall represents a small fraction of congenital ptosis patients [3]. The extent of eyelid movement during jaw-winking receives three classification levels, which include mild (<2 mm), moderate (2-4 mm), and severe (≥5 mm), and the majority of patients show moderate movements of their eyelids during jaw-winking according to Demirci et al. [4]. Patients with moderate to severe cases of synkinetic eyelid movement and ptosis need surgical intervention because their condition creates both cosmetic issues and visual axis obstruction.

Patients who have moderate to severe ptosis are often recommended to have a levator palpebrae superioris resection and a bilateral frontalis suspension [5]. The primary surgical goal for MGJWS consists of stopping the synkinetic eyelid movement known as “wink” and fixing the drooping eyelid. The various surgical techniques described for this condition demonstrate that experts have not established a standard optimal procedure. The standard treatment consists of disabling or removing the faulty levator muscle operation and attaching the eyelid to the frontalis muscle. The standard surgical procedure consists of unilateral levator excision or disinsertion alongside a frontalis sling procedure that utilizes fascia lata or a silicone rod. The sling procedure may be done unilaterally on the affected eye or bilaterally on both eyelids for symmetry, and the surgical techniques for levator manipulation include anterior and posterior approaches. The surgical procedures for MGJWS include partial levator muscle resections or plications to reduce wink amplitude while maintaining some levator function, direct levator-frontalis muscle anastomosis, and other innovative surgical methods. Each surgical method provides unique advantages and disadvantages regarding wink elimination and eyelid position in primary and downgaze positions, as well as complication rates and need for reoperation. The scarcity of MGJWS cases has led to case series-based publications instead of randomized trials until recent times. A systematic review was conducted to gather evidence from clinical trials starting from 2010 about MGJWS surgical treatments to evaluate quantitative results, complications, and recurrence rates for establishing optimal clinical practices. This review uses the Preferred Reporting Items for Systematic Reviews and Meta-Analyses (PRISMA) guidelines to select and screen studies and includes only clinical research findings from interventional trials and planned case series with surgical results during this time period.

## Review

Search strategy and study selection

**Figure 1:**
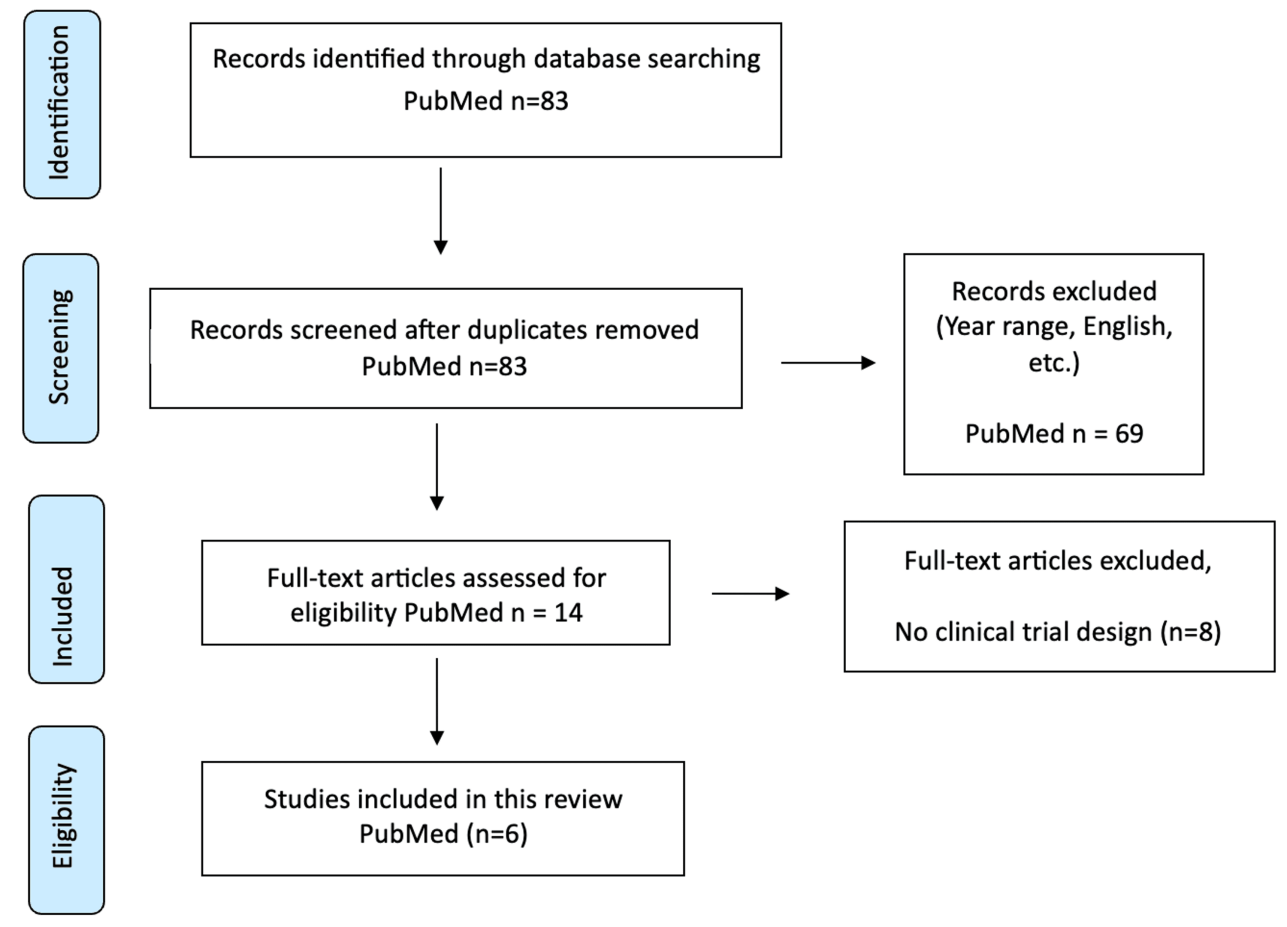
PRISMA flow diagram of literature search and study selection PRISMA, Preferred Reporting Items for Systematic Reviews and Meta-Analyses

A PubMed search for English-language studies containing “Marcus Gunn jaw-winking” was conducted with a filter from January 2010 until present. The initial search produced 83 records. The initial screening eliminated 69 records because they failed to satisfy the inclusion criteria and mainly consisted of case reports and review articles with non-surgical studies. The full-text assessment revealed 14 articles for potential inclusion. Eight studies failed to meet our requirements because they lacked a clinical trial design. The qualitative synthesis included six studies that were published after 2010. The PRISMA flow diagram presented in Figure 1 illustrates the search and selection process, along with exclusion reasons.

Characteristics of included studies

The six included studies investigated surgical interventions for MGJWS through clinical research. These articles each included patient populations between 10 and 48 individuals. The research base lacked randomized controlled trials and relied on observational studies, which include prospective and retrospective designs to provide quantitative outcome data, with a concentration on treating moderate to severe MGJWS patients who required surgical intervention for both ptosis repair and synkinesis treatment. Patients in the studies were primarily children and young adults whose ages spanned from childhood through their early twenties. Different studies tracked their participants in their follow-up between six months and five years.

The studies reported common outcome measures that included jaw-winking synkinesis resolution (defined by minimal eyelid movement during jaw motion), ptosis improvement through margin reflex distance (MRD1) or palpebral fissure height measurements, evaluation of eyelid symmetry in primary gaze and downgaze positions, patient satisfaction ratings, and assessment of complications and reoperation requirements. The definition of success varied across studies, but most required substantial wink reduction and satisfactory eyelid placement. The recorded complications included exposure keratopathy, lagophthalmos, and eyelid contour problems such as eyelash ptosis and loss of crease and sling-related problems. Ptosis residuals and wink recurrences that required additional surgical procedures were also reported. Table 1 presents a summary of the six included studies in the review, listed in order of chronological publication date.

**Table 1:** Included studies of surgical interventions for MGJWS (2010-2024) MGJWS, Marcus Gunn jaw-winking syndrome

Study (year)	Sample size	Surgical intervention	Outcomes	Complications/notes
Demirci et al. [4]	48 patients with moderate to severe MGJWS	Unilateral levator disinsertion/excision with frontalis suspension (26 bilateral and 4 unilateral)	Wink eliminated in 97% of patients; 87% had eyelid height symmetry within 1 mm in primary gaze and 80% in downgaze	10% eyelash ptosis, 10% loss of crease, and 3% entropion; low recurrence rate; follow-up ~5 years
Xiang et al. [6]	13 patients	Levator-frontalis anastomosis without sling	100% wink elimination; 77% achieved symmetric eyelid apertures; mild ptosis (<2 mm) in 23%	No major complications; follow-up ~12 months
Mandal et al. [7]	30 patients aged 7-40 years	Posterior-approach levator excision with unilateral silicone sling	Near-total wink elimination (mean residual, 0.4 mm); MRD1 improved to 4.0 mm; high satisfaction	No recurrence, no reoperations; minimal complications; follow-up ~1 year
Ning et al. [8]	42 patients (35 moderate/severe MGJWS)	Unilateral levator excision with frontalis suspension using silicone	100% wink elimination; fissure height ~7.9 mm post-op and symmetrical	1 case (2.9%) of wink recurrence; good overall cosmetic results; follow-up ~6 months
Bajaj et al. [9]	10 patients	Levator plication (levator tightening, not excision)	30% had complete wink resolution and 70% had residual but reduced wink; 90% achieved satisfactory ptosis correction	No lid lag, crease loss, or exposure issues; follow-up ~6-12 months
Shah et al. [10]	23 patients	Unilateral tarso-frontalis sling with silicone rod and no levator disinsertion	No severe wink post-op; 87% had mild residual wink and 13% moderate; all had adequate eyelid elevation	55% required sling tightening; no cases of keratopathy; follow-up ~7 months

Surgical outcomes

The two-stage procedure of levator excision with frontalis suspension is the most frequently described intervention and usually shows good results. Demirci et al. found that the surgical procedure that removed the levator muscle with a frontalis sling eliminated jaw-winking completely in 97% of patients, although one patient (3%) maintained small synkinetic flicker movements (6 mm pre-op to 2 mm) [4]. The 2019 research conducted by Ning and colleagues produced 100% elimination of wink occurrences through unilateral levator excision and sling implantation [8]. The studies revealed that ptosis results were outstanding because postoperative lid elevation functioned within 1 mm of the contralateral lid in primary gaze in more than 85-88% of patients [4]. Long-term results from Demirci et al.’s study revealed that ptosis and synkinesis did not return significantly during the mean follow-up period of five years [4]. The surgical outcomes of Demirci et al. and Ning et al. showed that nearly all patients achieved identical eyelid placement in primary gaze, and most patients also displayed good eye symmetry while looking downward, especially when bilateral sling procedures were used [4,8]. Demirci et al. also reported that among their 30 surgical patients, 26 patients received frontalis suspension in both eyes, which led to excellent downgaze symmetry (88% within 1 mm) [4]. The patients who received a unilateral sling experienced perfect symmetry in primary gaze but achieved downgaze symmetry in only 25% of cases [4]. The procedure of bilateral frontalis suspension creates full symmetry of eye movement but results in the loss of normal eyelid depression during downgaze to maximize symmetry. The procedure that involves unilateral suspension does not need surgery on the healthy eye but leads to unsymmetrical eyelid movement during downgaze. The literature therefore shows that levator excision with frontalis sling (especially bilateral sling when needed) provides excellent results in eliminating jaw-wink and correcting ptosis in moderate to severe MGJWS patients [4,8]. Patient and parent satisfaction with the cosmetic outcome of this treatment approach was high according to both studies. 

Levator muscle weakening vs. preservation approaches

One issue of contention is whether or not the levator has to be totally excised in order to prevent the synkinesis, or if more conservative procedures are enough. The conventional teaching is that any residual levator function would enable the wink to persist, and so many surgeons recommend complete levator excision. However, a modified levator plication technique was described by Bajaj et al. to treat MGJWS without having to remove the levator [9]. In this small series (10 eyelids), the levator aponeurosis was plicated to correct ptosis, instead of being excised. This improved the ptosis in 90% of cases (there was a significant increase in MRD1 by ~2.4 mm) and was also anatomically less destructive. Bajaj et al. reported that there were no cases of postoperative lagophthalmos, lid crease loss, or lid contour abnormalities that sometimes occur after levator excision [9]. The price paid was that the wink was not eradicated in most of the patients; only 30% had no wink at all, while the rest had a wink, but a much weaker one [9]. Thus, levator plication may reduce the synkinesis and improve appearance in mild to moderate MGJWS, but it is not as effective for complete wink elimination. The authors proposed that this technique might be used for patients in whom a small amount of wink is acceptable in order to potentially decrease the risk of complications. However, larger series suggest that true levator excision is often necessary to eliminate the abnormal movement altogether [4].

Another innovative way that tries to get rid of wink and at least some dynamic elevation is levator-frontalis muscle anastomosis. Xiang et al. described this technique in 13 patients, in whom the levator muscle was detached from the tarsus, and instead of discarding it, it was directly attached to the frontalis muscle [6]. In effect, this is a dynamic eyelid suspension without a sling material; the frontalis contraction will lift the lid, and the levator will be used as a static crutch (thus eliminating the aberrant innervation in the process). Xiang et al. reported 100% resolution of jaw-winking with this method, with 77% of patients having symmetric lid apertures and only a few cases of mild ptosis (≤2 mm) [6]. No notable complications were observed. This result is practically identical to those of levator excision + sling. This anastomosis technique has the benefit of not requiring fascia lata or silicone implants (useful in low-resource settings or when autologous fascia is not available). It effectively “obliterates” the function of the levator by redirecting it, thus preventing the wink. However, this procedure may be difficult to perform, and not many surgeons may have adopted it; further data and follow-up are required to confirm its long-term efficacy. Nonetheless, this is proof of concept that synkinetic innervation does not have to be eliminated by removing the muscle entirely, but by redirecting it to the frontalis.

The most non-invasive surgical choice is frontalis suspension alone (without levator excision). In theory, simply placing a sling to lift the eyelid may decrease the amount of eyelid movement with jaw action, even if the levator is left intact. Shah et al. investigated this treatment strategy in 23 patients who received a unilateral tarso-frontalis silicone sling without touching the levator muscle [10]. Postoperatively, none of the patients had a severe jaw-wink anymore, but none had complete elimination of the wink either. In fact, 87% still had a mild residual wink (<2 mm excursion), and 13% had a moderate residual wink [10]. In essence, the synkinetic movement was “dampened” by the sling: the eyelid, now tethered to the forehead, could not jerk upward as dramatically with jaw movement, but some movements still persisted in most cases. On the positive side, ptosis was adequately corrected in all patients for primary gaze (the sling elevates the lid). This technique also avoided any manipulation of the levator muscle, thus making the surgery simpler and theoretically decreasing the risk of eyelid contour problems. Shah et al. noted that there were no cases of significant exposure keratopathy in spite of poor Bell’s phenomenon in some patients, and in a few cases, mild residual ptosis may have even protected the cornea [10]. However, a notable drawback was that more than half of the patients needed re-adjustment or tightening of the sling at follow-up due to sling slackening (some late ptosis recurrence). This shows that a sling-only approach may not be a one-and-done, durable solution. In summary, unilateral frontalis sling without levator excision can markedly decrease the severity of the jaw-wink, making it mild and more socially acceptable, but it usually does not eliminate the synkinesis. Patients must be counseled that a small wink might remain. This approach may be considered in moderate cases or if one wants to avoid more aggressive surgery, but most surgeons reserve it for special circumstances, given the availability of more definitive procedures. 

Posterior approach vs. anterior approach

The research by Mandal et al. presented a technical adjustment instead of a completely new procedure [7]. Mandal and colleagues performed the levator excision via a posterior approach through the conjunctiva, identifying Whitnall’s ligament in contrast to the traditional anterior approach through the eyelid crease [7]. The authors chose this approach to eliminate all levator muscle fibers near Whitnall’s (the aberrant synkinetic nerve entry point) while minimizing anterior scarring. The silicone sling method produced outstanding functional results in their prospective study of 30 patients since no patient developed jaw-winking recurrence and no patient needed additional surgery [7]. The post-operative measurements of MRD1 and palpebral fissure showed substantial improvements (mean MRD1 ~4 mm post-op). The remaining wink amplitude measured almost zero in all patients. The authors stressed that lid height symmetry in primary gaze could be achieved unilaterally without performing surgery on the opposite lid. The non-bilateral surgical approach stands as a main advantage of their method since surgeons previously performed bilateral sling procedures for symmetry improvement, but Mandal et al. achieved satisfactory unilateral results, thus avoiding surgery on the normal eye [7]. The study reported minimal complications since no patient developed reattachment of the levator or recurrent synkinesis during one year of follow-up [7]. The study results indicate that precise posterior excision produces results that might be better than the standard technique, even though a direct anterior approach comparison was absent. 

The majority of patients achieve complete elimination of jaw-winking through strategies that eliminate levator function by excision or anastomosis. The main purpose of frontalis suspension (sling) is to correct ptosis, and surgeons perform this procedure either unilaterally or bilaterally to achieve the desired balance between primary gaze and downgaze symmetry. The interventions that include levator excision (or its equivalent) produce the highest synkinesis resolution rates above 95%, but levator-sparing methods result in some remaining wink. All surgical approaches result in good ptosis outcomes because various techniques can lift the eyelid, yet the main distinction exists between complete wink elimination and wink reduction. Demirci et al. and Mandal et al. show that patients experience the highest satisfaction when their wink disappears completely because of the significant cosmetic enhancement [4,7]. Patients who have mild MGJWS or who prefer minimal surgical intervention can achieve significant quality of life improvement through wink reduction procedures.

Complications and recurrence

Current clinical trials demonstrate that surgical correction of MGJWS leads to good tolerance from patients with minimal severe complications. The studies show that large ptosis surgeries result in minimal exposure keratopathy and lagophthalmos occurrences. Demirci et al. and Shah et al. both reported no cases of exposure keratopathy that needed surgical intervention, although Shah et al. specifically mentioned their sling-only patients maintained perfect exposure despite some patients showing a poor Bell’s reflex [4,10]. Children make up most MGJWS patients because their corneas can safely handle small exposure incidents, and preoperative amblyopia represents a greater concern than postoperative amblyopia. The upper lid cannot descend normally after sling placement, which results in lid lag in downgaze, together with expected lid lag. The patients undergoing unilateral sling procedures developed downgaze lid lag as a designed outcome according to Shah et al. [10]. The surgical outcome represents an expected result rather than a complication since bilateral sling procedures prevent relative lag between eyes but reduce the downgaze mobility of both lids. Postoperative lash ptosis appeared in 10% of patients along with superior eyelid crease loss in 10% of patients, as documented by Demirci et al. [4]. The observed problems are minor, and most cases require either small surgical adjustments or represent only cosmetic issues. The tissue-sparing plication technique developed by Bajaj et al. maintained the eyelid crease, while the anterior approach levator excision resulted in crease disruption [9]. The incidence of entropion stands at 3% according to Demirci et al., but this condition has become rare in current studies, especially when silicone slings are used because they remain elastic [4]. Similarly, Sthapit and Saiju documented that patients who underwent excision with sling surgery achieved complete wink resolution while experiencing only mild side effects, including lid lag and one instance of overcorrection, which confirmed the procedure’s safety and effectiveness [11].

The recurrence of true aberrant jaw-winking (reinnervation or regrowth of the levator, causing synkinesis to return) is very rare when the levator is adequately dealt with. None of the 26 bilateral sling patients had recurrence of wink, and only one of the four unilateral sling patients had a mild residual wink in Demirci et al.’s long-term series [4]. Ning et al. similarly found a 2.9% recurrence rate of jaw-wink (one patient) after unilateral excision + sling, attributing it to incomplete initial removal of levator fibers [8]. Mandal et al. had zero recurrences at the one-year follow-up with their thorough posterior excision approach [7]. Those approaches that do not remove the levator inherently leave some wink, but that is an expected residual rather than a “recurrence” [7,8]. In those cases, if the remaining wink is bothersome, a second-stage levator excision can be performed (two patients in Shah et al.’s series eventually underwent a levator disinsertion later to fully abolish the wink) [10]. Reoperation rates were modest: aside from sling adjustments (which were needed in >50% of Shah et al.’s patients due to sling loosening), most series saw around 10-15% of patients undergoing a secondary procedure, usually for ptosis fine-tuning. For example, Demirci et al. noted that three of 30 operated patients (10%) needed a second surgery for ptosis under-correction [4]. This tends to occur if initial sling tension is inadequate or if the fellow eye’s condition (e.g., amblyopia treatment and strabismus) changes the lid height requirements. Importantly, even secondary revisions generally achieved the desired outcome; none of the studies indicated any “failures” where satisfactory lid position and wink elimination could not eventually be attained.

Bilateral MGJWS

Bilateral MGJWS is a rare clinical phenomenon that is not well described in the literature, with only a few case reports and small series described by Sobel and Allen [12]. In bilateral MGJWS, both eyelids exhibit a wink phenomenon that is synkinetic, but may be asynchronous or to a greater or lesser degree, because each levator may have an aberrant innervation from trigeminal fibers. This condition is especially difficult to manage surgically. On the one hand, because bilateral involvement is virtually certain, surgeons will usually proceed with bilateral levator excision/weakening and bilateral frontalis suspension; there is no normal eye to save. The goal is to stop the wink on both sides and to lift both lids up. In fact, the results of the very few bilateral cases that have been reported seem to suggest that symmetry is actually easier to obtain than in unilateral cases since both eyelids are being set to a new position (Bowyer and Sullivan reported that bilateral levator excision + sling eliminated winking in all their bilateral cases) [13]. Both lids will have no downward motion and no blink strength after surgery, so the surgeon must be careful of postoperative exposure keratopathy, and therefore, aggressive lubrication and protective measures should be instituted in the immediate postoperative period for bilateral cases. Another consideration is that some bilateral MGJWS patients have ptosis primarily in one eye (the other eye may wink but not have significant ptosis). Quaranta-Leoni et al. mentioned at least one patient in their series with bilateral MGJWS and unilateral ptosis; such cases might be treated by surgery on the ptotic side (to correct ptosis and wink) and observation of the other side if the wink is mild [14]. However, truly bilateral and symmetric MGJWS with ptosis in both eyes should be treated with a symmetric surgical approach to prevent a see-saw effect. On the other hand, bilateral MGJWS does not appear to be as rare as previously thought. Sobel and Allen contend that the earlier literature made it out to be a curiosity, but careful review reveals that a small percentage (~2-3%) of MGJWS cases could have bilateral involvement [12]. Nevertheless, all published bilateral cases have been managed with the standard techniques, and the outcomes do not differ significantly, except for the need for increased awareness of the exposure complications. There is no evidence that bilateral cases have any higher recurrence rate; if anything, removing both levators mostly ensures that there will be no wink left. Each bilateral case should be treated separately; in some cases of asymmetry, it may be possible to operate on the worse eye first, but in the end, most of them will require bilateral treatment to achieve optimal symmetry.

## Conclusions

Surgical intervention for MGJWS is highly effective in improving ptosis and eliminating the jaw-wink synkinesis, thereby significantly enhancing patients’ visual function and social confidence. A systematic review of clinical studies published since 2010 finds that levator excision (or full levator inactivation) combined with frontalis suspension remains the most suitable approach for moderate to severe MGJWS, yielding near-complete abolishment of the wink in ~95-100% of cases and excellent eyelid position outcomes. Bilateral or unilateral frontalis sling operations should be performed according to the needs of each patient: bilateral sling procedures produce superior symmetry during downgaze, but unilateral slings prevent surgical manipulation of the normal eye and produce some asymmetry in downgaze. New surgical techniques like levator-frontalis anastomosis are being investigated as alternatives to traditional excision, and levator plication or sling-only procedures are being used in select cases, but they generally only reduce synkinesis rather than eliminate it.
